# Obesity paradox as a new insight from postoperative complications in gastric cancer

**DOI:** 10.1038/s41598-023-36968-7

**Published:** 2023-06-21

**Authors:** Hajime Kamiya, Shuhei Komatsu, Keiji Nishibeppu, Takuma Ohashi, Hirotaka Konishi, Atsushi Shiozaki, Takeshi Kubota, Hitoshi Fujiwara, Eigo Otsuji

**Affiliations:** Division of Digestive Surgery (Gastric Surgery Division), Department of Surgery, Kyoto Prefectural University of Medicine, 465 Kawaramachi-hirokoji, Kamigyo-ku, Kyoto, 602-8566 Japan

**Keywords:** Cancer, Oncology

## Abstract

The obesity paradox is reported to exist in various diseases. However, obesity is a pivotal issue in gastric cancer (GC) patients because of the surgical difficulty related to postoperative abdominal infectious complications (PAIC). This study clarified the existence of the obesity paradox in GC. Between 1997 and 2015, 1536 consecutive patients underwent curative gastrectomy. Of all patients, 18.6% (285/1536) were obese and tended to have a better prognosis (*P* = 0.073). In patients without PAIC, obesity was a significant prognostic factor for 5-year overall survival (*P* = 0.017). PAIC was an independent poor prognostic factor in both obese and non-obese patients (*P* < 0.001; hazard ratio [HR] 4.22 and 1.82). In pStage II–III patients, there was a large and significant prognostic difference between non-PAIC and PAIC obese patients (*P* = 0.006; 5-year overall survival: 69.7% vs. 43.8%) related to the higher incidence of peritoneal recurrence in PAIC obese patients (*P* = 0.035; 31% vs. 10%). Whereas, there was a small prognostic difference between non-PAIC and PAIC non-obese patients (*P* = 0.102; 5-year overall survival: 56.5% vs. 51.9%). Although the obesity paradox is present in GC, PAIC had a more negative prognostic impact through peritoneal recurrence in obese GC patients.

## Introduction

Gastric cancer (GC) is the fifth most common cancer and the third most common cause of death worldwide^1–3^. Recent developments in diagnostic technology, evidence-based surgical procedures, novel chemotherapy, and immunotherapy have enhanced detection rates and drastically improved the early and long-term outcomes for GC patients. However, GC remains a serious health problem with various clinical issues^4–7^. In recent years, the obesity rate has been increasing worldwide because of the westernization of lifestyle, especially diet^8–10^. Obesity is a major cause of various diseases such as cardiovascular disease and metabolic disease. However, various studies have identified that obese patients have a better prognosis compared to underweight patients in several chronic diseases, and this phenomenon is known as the obesity paradox^11,12^.

In GC patients, obesity has had a negative impact on surgical procedures, leading to increased blood loss, poor lymph node dissection, and postoperative complications^13–15^. In addition, because postoperative complications lead to a poor oncological prognosis^16–20^, obese GC patients may have poor prognoses. Therefore, the existence and mechanisms of the obesity paradox in GC remain controversial^21^.

This study clarified whether the obesity paradox is present in GC from the viewpoint of postoperative complications. Our results may demonstrate the positive impact of obesity on the prognoses of patients with GC.

## Results

### Clinicopathological characteristics of obese GC patients

There were 285 obese GC patients among 1536 consecutive patients with GC. A comparison of clinicopathological factors between obese and non-obese patients with GC is presented in Table 1. The obese group was significantly associated with a higher incidence of males (*P* = 0.004; 74.4% vs. 65.6%), cardiovascular disease (*P* < 0.001; 38.2% vs. 26.9%), and endocrine diseases (*P* = 0.008; 21.8% vs. 15.1%) than the non-obese group. There was no significant difference in PAIC rate between obese and non-obese patients (10.9% (31/285) vs. 8.7% (108/1251)) or between other surgical factors.

**Table 1:** Clinicopathological characteristics of obese and non-obese gastric cancer patients.

Variables	Total	Obese patients (n = 285)	Non-obese patients (n = 1251)	*P* value
Gender				
Female	503	73 (26%)	430 (34%)	**0.004**
Male	1033	212 (74%)	821 (66%)	
Age				
< 75	1194	220 (77%)	974 (78%)	0.813
≥ 75	342	65 (23%)	277 (22%)	
Albumin (g/dl)				
< 3	168	25 (9%)	143 (11%)	0.208
≥ 3	1368	260 (91%)	1108 (89%)	
Hemoglobin (g/dl)				
< 8	115	20 (7%)	95 (8%)	0.804
≥ 8	1421	265 (93%)	1156 (92%)	
Cardiovascular disease				
( − )	1091	176 (62%)	915 (73%)	** < 0.001**
( +)	445	109 (38%)	336 (27%)	
Digestive disease				
( − )	1137	219 (77%)	918 (73%)	0.261
( +)	399	66 (23%)	333 (27%)	
Endocrine and autoimmune disease				
( − )	1285	223 (78%)	1062 (85%)	**0.008**
( +)	251	62 (22%)	189 (15%)	
Hepatic disease				
( − )	1442	269 (94%)	1173 (94%)	0.785
( +)	94	16 (6%)	78 (6%)	
Neurological disease				
( − )	1460	277 (97%)	1183 (95%)	0.069
( +)	76	8 (3%)	68 (5%)	
Renal and urologic disease				
( − )	1498	281 (99%)	1217 (97%)	0.289
( +)	38	4 (1%)	34 (3%)	
Respiratory disease				
( − )	1472	275 (97%)	1197 (96%)	0.624
( +)	64	10 (3%)	54 (4%)	
p Stage				
I	1037	195 (68%)	842 (67%)	0.779
II + III	499	90 (32%)	409 (33%)	
Surgical approach				
Open	1051	198 (70%)	853 (68%)	0.724
Lap	485	87 (30%)	398 (32%)	
Lymphadenectomy				
D2 ≤	707	126 (44%)	581 (46%)	0.511
< D2	829	159 (56%)	670 (54%)	
PAIC^a^				
( − )	1396	254 (89%)	1142 (91%)	0.255
( +)	140	31 (11%)	109 (9%)	

^a^Postoperative abdominal infectious complications: anastomotic leakage, pancreatic fistula, and intra-abdominal abscess in grade II or higher of Clavien–Dindo classification.

### Multivariate analyses using the Cox’s proportional hazard model in GC patients with or without obesity

Table 2 shows the prognostic factors in patients undergoing curative gastrectomy for GC according to obesity status. In non-obese patients, multivariate analyses using Cox’s proportional hazards model revealed that age ≥ 75 years (*P* < 0.001; hazard ratio [HR] 2.09), advanced pathological status of pStage II or high (*P* < 0.001; HR 5.42), open gastrectomy (*P* < 0.001; HR 3.79), radical D2 lymphadenectomy (*P* < 0.001; HR 1.95), and PAIC (*P* < 0.001; HR 1.82) were independent poor prognostic factors. In obese patients, however, advanced pathological status (*P* < 0.001; HR 5.99) and PAIC (*P* < 0.001; HR 4.22) were independent poor prognostic factors. PAIC was an independent poor prognostic factor in both obese and non-obese patients, but PAIC was a stronger factor in obese patients.

**Table 2:** Multivariate analyses of survival after surgery using the Cox’s proportional hazard model according to obesity status.

				Obese patients					Non-obese patients				
				Multivariate ^a^					Multivariate ^a^				
				HR ^b^	95% CI ^c^			*P* value	HR ^b^	95% CI ^c^			*P *value
Age	75 ≤	versus	< 75						2.09	1.58	–	2.76	< 0.001
pStage	II + III	versus	I	5.99	2.96	–	12.12	< 0.001	5.42	3.92	–	7.49	< 0.001
Surgical approach	Open	versus	Lap						3.79	2.35	–	6.11	< 0.001
Lymphadenectomy	D2 ≤	versus	< D2						1.95	1.43	–	2.65	< 0.001
PAIC ^d^	( +)	versus	( − )	4.22	2.11	–	8.44	< 0.001	1.82	1.26	–	2.62	< 0.001

^a^ Multivariate survival analysis was performed using Cox’s proportional hazard model.

^b^ HR: Hazard ratio.

^c^ CI: Confidence interval.

^d^ Postoperative abdominal infectious complications: anastomotic leakage, pancreatic fistula, and intra-abdominal abscess in grade II or high of Clavien–Dindo classification.

### Overall survival curves according to the combination of the PAIC and obesity status

**Figure 1:**
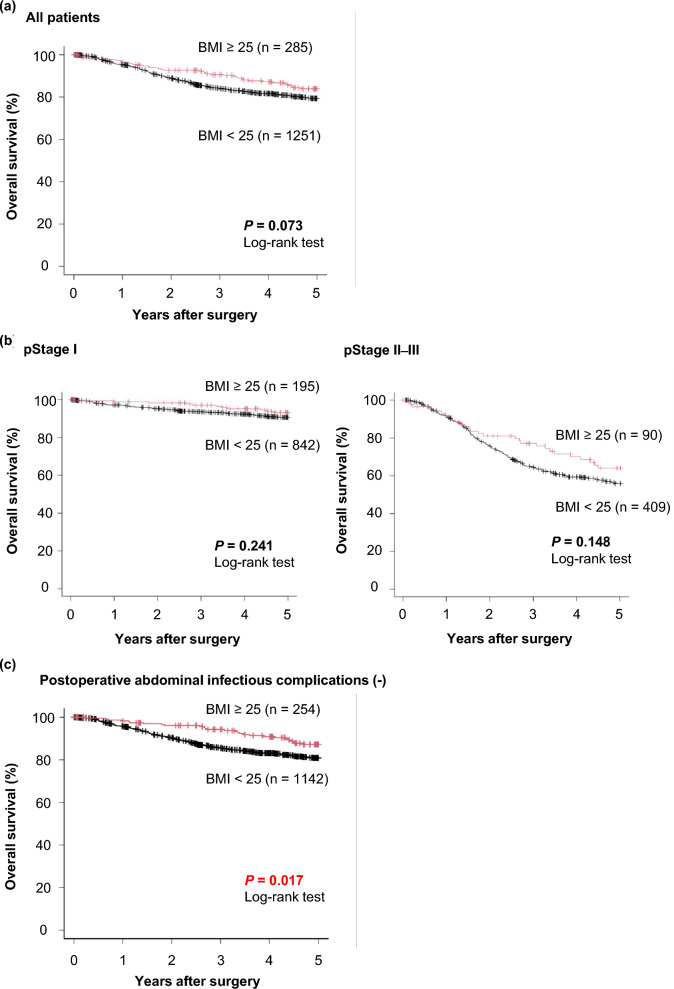
(**a**) Survival curves for all patients. There was no prognostic difference between obese and non-obese patients (*P* = 0.073). (**b**) Survival curves according to pStage. There was no prognostic difference between obese and non-obese patients (*P* = 0.241, *P* = 0.148). (**c**) Survival curves for patients without postoperative abdominal infectious complications (PAIC) according to a BMI cut-off value of 25. Obese patients had a significantly better prognosis than non-obese patients (*P* = 0.017).

**Figure 2:**
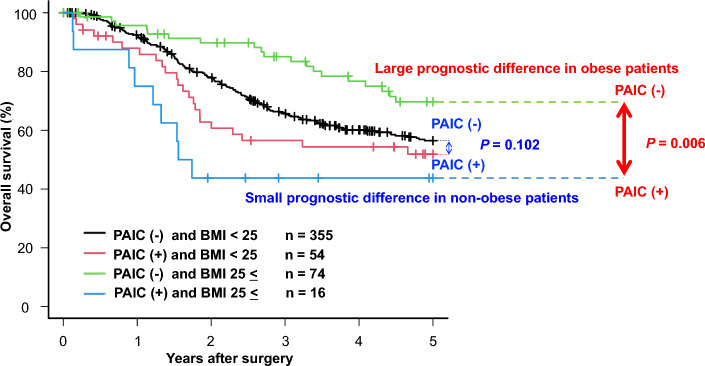
Overall survival curves according to the combination of PAIC and obesity status. In 499 consecutive patients with pStage II–III GC, there was a large and significant prognostic difference between non-PAIC and PAIC obese patients (*P* = 0.006; 5-year overall survival: 69.7% vs. 43.8%), related to the higher incidence of peritoneal recurrence in PAIC obese patients (*P* = 0.035; 31.2% vs. 9.5%). In contrast, there was a small prognostic difference between non-PAIC and PAIC non-obese patients (*P* = 0.102; 5-year overall survival: 56.5% vs. 51.9%).

Next, we evaluated the prognostic effects of obesity and PAIC on survival curves. In all patients, there was no prognostic difference between obese and non-obese patients (Fig. 1a) although obesity tended to be a better prognostic factor (*P* = 0.073). Also, when patients were classified as early pStage (pStage I) or advanced pStage (pStage II–III), there was no difference in prognosis between obese and non-obese patients (Fig. 1b). However, obese patients had a significantly better prognosis than non-obese patients among patients without PAIC (*P* = 0.017), suggesting the obesity paradox (Fig. 1c). Moreover, the prognostic difference caused by PAIC was small in the non-obese patients with pStage II–III GC (*P* = 0.102; 5-year overall survival: 56.5% vs. 51.9%), whereas obese patients with PAIC had a significantly extremely poorer prognosis than those without PAIC (*P* = 0.006; 5-year overall survival: 69.7% vs. 43.8%) (Fig. 2).

### Recurrence patterns and rates in patients with pStage II–III GC according to the combination of obesity and PAIC

**Figure 3:**
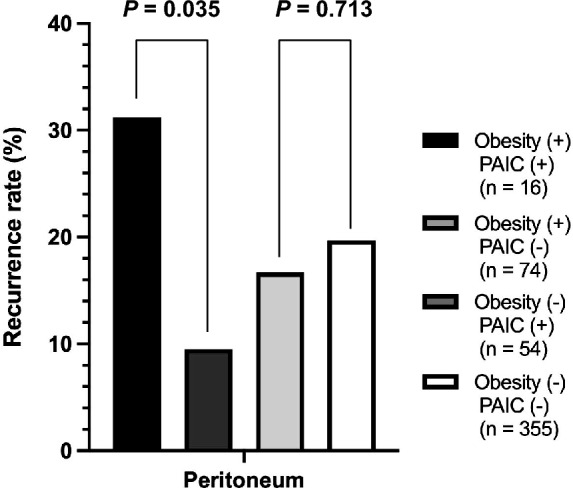
Peritoneal recurrence rates in patients with pStage II–III GC according to the combination of obesity and PAIC. In 499 consecutive patients with pStage II–III GC, obese patients with PAIC were more likely to relapse with peritoneal recurrence than patients without PAIC (*P* = 0.035; 31.2% vs. 9.5%).

Finally, we investigated the prognostic impact of obesity and PAIC with advanced pStage II–III GC. Obese patients with PAIC were more likely to relapse with peritoneal recurrence than patients without PAIC (*P* = 0.035; 31% vs. 10%). In contrast, there was no significant correlation between PAIC and peritoneal recurrence in non-obese patients (*P* = 0.713; 17% vs. 20%) (Table 3** and **Fig. 3).

**Table 3:** Recurrence patterns and rates in patients with pStage II–III gastric cancer according to the combination of obesity and PAIC.

Recurrence	Obesity ( +) PAIC ( +) (n = 16)	Obesity ( +) PAIC (-) (n = 74)	*P* value	Obesity (-) PAIC ( +) (n = 54)	Obesity (-) PAIC (-) (n = 355)	*P*-value
Total patients	7 (44%)	22 (30%)	0.376	20 (37%)	129 (34%)	0.882
Recurrence types						
Peritoneum	5 (31%)	7 (10%)	**0.035**	9 (17%)	70 (20%)	0.713
Local	1 (6%)	2 (3%)	0.448	1 (2%)	11 (3%)	1.000
Lymph node	1 (6%)	7 (10%)	1.000	4 (7%)	34 (10%)	0.802
Hematogenous	2 (13%)	10 (14%)	1.000	9 (17%)	37 (10%)	0.162
Others	2 (13%)	1 (1%)	0.326	0 (0.0%)	9 (3%)	0.614

### Biochemical mechanisms affecting poor prognosis in obese patients with postoperative complications

**Figure 4:**
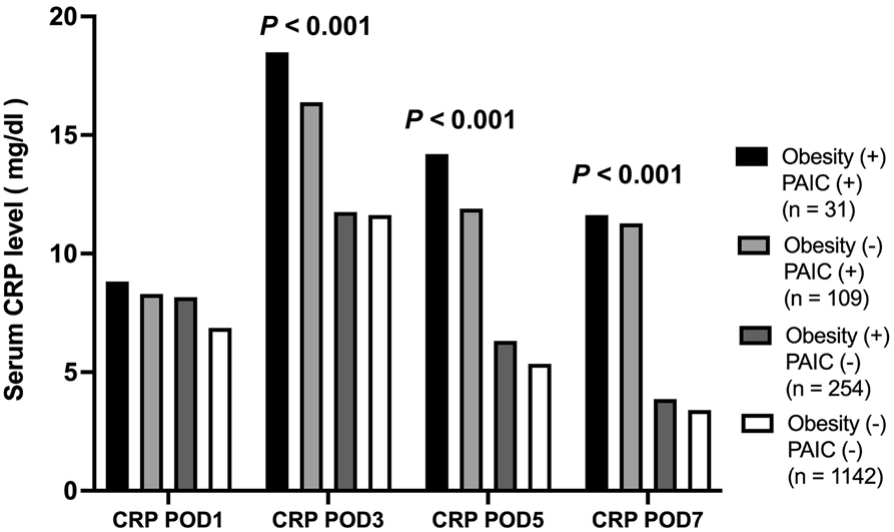
Comparison of postoperative serum CRP level according to the combination of PAIC and obesity status. Obese patients with PAIC had significantly higher serum CRP level than obese patients without PAIC, non-obese patients with PAIC, and non-obese patients without PAIC (P < 0.001; 18.5 vs. 16.4 vs. 11.8 vs. 11.6 mg/L) on postoperative day 3. The results were similar on postoperative day 5 (P < 0.001; 14.2 vs. 11.9 vs. 6.3 vs. 5.4 mg/L) and day 7 (P < 0.001; 11.6 vs. 11.3 vs. 3.9 vs. 3.4 mg/L).

Next, we examined the causes of the poor prognosis in obese patients with PAIC from the viewpoint of biochemical analysis. We focused on postoperative serum CRP level according to the combination of obesity and PAIC. Obese patients with PAIC had a significantly higher serum CRP level than obese patients without PAIC, non-obese patients with PAIC, and non-obese patients without PAIC (*P* < 0.001; 18.5 vs. 16.4 vs. 11.8 vs. 11.6 mg/L) on postoperative day 3. We observed similar results on postoperative day 5 (*P* < 0.001; 14.2 vs. 11.9 vs. 6.3 vs. 5.4 mg/L) and day 7 (*P* < 0.001; 11.6 vs. 11.3 vs. 3.9 vs. 3.4 mg/L) (Fig. 4).

### Prognostic analysis and recurrence patterns using a cohort after propensity score matching

Finally, in order to exclude selection bias from potential confounders, we analyzed the prognosis and recurrence patterns using a cohort after propensity score matching. After propensity score matching, there were no significant differences in clinicopathological characteristics between obese and non-obese patients (Supplemental Table 1). The results of prognosis and recurrence patterns were approximately similar to those before propensity score matching. There was no prognostic difference between obese and non-obese patients (Supplemental Fig. 1a, b, c). However, obese patients had a significantly better prognosis than non-obese patients among patients without PAIC (*P* = 0.032) (Supplemental Fig. 1d). The prognostic difference caused by PAIC was larger in the obese patients than non-obese patients (Supplemental Fig. 2) and was a stronger prognostic factor in obese patients than non-obese patients (HR 4.22 vs. 2.54) (Supplemental Table 2). In recurrence pattern, obese patients with PAIC were more likely to have peritoneal recurrence than those without PAIC. In contrast, there was no significant correlation between PAIC and peritoneal recurrence in non-obese patients (*P* = 1.000) (Supplemental Table 3).

## Discussion

The obesity paradox has been reported in various diseases owing to better nutrition, immunity, and tolerability in treatments. However, it has been unclear and controversial whether the obesity paradox exists in GC. This is because obesity is a pivotal issue of GC patients, affecting the surgical difficulty and postoperative abdominal infectious complications (PAIC), and inducing poor long-term survival. This study clearly demonstrated the obesity paradox in GC patients, particularly in those without PAIC. PAIC might affect a stronger and more negative prognostic impact in obese GC patients through peritoneal recurrence following gastrectomy.

The obesity paradox was suggested more than 20 years ago and is a well-known phenomenon in various diseases. Initial studies identified that being overweight could be a favorable prognostic factor in cardiovascular and metabolic diseases^11,12^. In solid cancers, several studies have identified that overweight patients have a better prognosis compared to normal-weight patients^22–25^. In esophageal cancer, Kayani et al. demonstrated that obesity was not a risk factor for postoperative complications and that obese patients have better prognoses than non-obese patients^22^. In colorectal cancer, Walter et al. have also reported that the obesity paradox was present in obese and smaller weight loss patients^23^. In renal cell carcinoma, Hakimi et al. reported that obese patients had a low malignant tumor compared to non-obese patients^24^. Moreover, in lung cancer, Kichenadasse et al. reported that overweight non-small cell lung cancer patients had a better prognosis and were more responsive to immune checkpoint inhibitor therapy^25^. Thus, the obesity paradox could be more likely to exist in various cancers, including gastrointestinal cancers.

Until now, there has been no clear evidence of whether obesity is associated with a better or worse prognosis in patients with GC. This is because obesity has been a negative factor for surgical procedures leading to increased blood loss, poor lymph node dissection, and postoperative complications^13–15^. Therefore, it has been controversial whether or not the obesity paradox exists. According to the WHO World Health Survey, Japan, South Korea, and Poland have relatively low obesity rates compared to Western countries^9^. In these countries which have fewer postoperative complications, some studies suggested that obesity was a better prognostic factor^26–28^. On the other hand, several studies from Western countries suggested that obesity was a poor prognostic factor^29^. Western countries have a higher incidence of obesity, which is associated with the difficulty of surgery and potentially increased postoperative complications. Thus, these differences might affect whether the prognostic effect of obesity was better or not in GC patients.

In this study, we demonstrated the striking findings that there was a strong and significant prognostic difference between non-PAIC and PAIC obese patients (*P* = 0.006; 5-year overall survival: 69.7% vs. 43.8%), related to the higher incidence of peritoneal recurrence in PAIC obese patients (*P* = 0.035) with advanced pStage II–III. In contrast, there was a small prognostic difference between non-PAIC and PAIC non-obese patients (*P* = 0.102; 5-year overall survival: 56.5% vs. 51.9%).

Adipocytes may be associated with the mechanism of the obesity paradox in GC because they control metabolism and immune interplay relevant to energy homeostasis^30^, thus contributing to a favorable prognosis. However, adipocytes also produced various hormones and adipokines such as HMGB-1^31^. HMGB-1 has been identified as a proinflammatory mediator and is expressed twofold more in adipose tissue from obese compared to normal-weight individuals^32^. It was reported to interact with the NF-κB pathway to initiate the transcription of various proinflammatory cytokines including TNF-α, IL-6, and IL-1b to promote inflammation^33,34^. In clinical settings, Osoegawa et al. demonstrated that higher surgical invasion increased the concentration of postoperative plasma HMGB-1^35^. In our study, serum CRP level, which was the result of inflammation induced by these adipokines, reproduced a similar phenomenon. CRP can be measured more easily than adipokines and reflect the level of postoperative inflammation. We have reported in previous studies that high postoperative CRP level is correlated with obesity and contributes to a poor prognosis^36,37^. These findings also suggested that obese patients have potentially more adipokines such as HMGB-1 and potentially have a poorer prognosis if postoperative complications occur.

The present study was limited by its retrospective design and small cohort. Additionally, the prolonged recruitment period over which the retrospective analysis was performed at a single institution may have been influenced by possible variations in treatment strategies over time. Therefore, a prospective observational study with several large cohorts or a nationwide clinical database study may be needed to validate the existence of the obesity paradox in GC. In conclusion, the obesity paradox could be present in GC patients without PAIC. However, PAIC might affect a negative impact in obese GC patients through peritoneal recurrence following gastrectomy.

## Methods

### Patients and procedures

This study was institutionally approved by the Kyoto Prefectural University of Medicine, and each participant provided written informed consent. A total of 1536 patients who underwent curative surgery for GC at our institute between 1997 and 2015 were included in this study. Furthermore, we retrospectively analyzed the clinicopathological features and prognostic outcomes. Finally, we evaluated the compatibility of our findings with the 8th edition of the AJCC/UICC TNM classification system.

The postoperative follow-up program at our institution comprises a regular physical examination as well as laboratory blood tests and chest X-rays every three or six months. When possible, endoscopy and ultrasonography, or computed tomography, were performed annually for the first five years. All enrolled patients underwent pathological or macroscopic curative resection (R0). Histological types were classified as differentiated (papillary adenocarcinoma, or moderately or well-differentiated adenocarcinoma) or undifferentiated (poorly differentiated or undifferentiated adenocarcinoma, signet-ring cell carcinoma, or mucinous adenocarcinoma) based on the 15th edition of the Japanese Classification of Gastric Carcinom^38^.

Considering the selection bias from potential confounders, propensity score-matched analysis was performed to compare the outcomes between obese patients and non-obese patients. The variables for calculating the propensity score were gender, age at surgery, presence of cardiovascular disease, presence of endocrine and autoimmune disease, presence of neurological disease, pStage and presence of PAIC, that were clinically important and tended to difference in clinicopathological characteristics between obese and non-obese patients.

### Definition of obesity and postoperative abdominal infectious complications

In this study, we defined obese patients as BMI 25 or higher as a previous report^39^. Regarding the postoperative complications, we focused on PAIC, because previous major studies identified that PAIC has a strong impact on poor prognosis in GC^17^. In this study, we defined PAIC as anastomotic leakage, pancreatic fistula, and intra-abdominal abscess in grade II or higher of the Clavien–Dindo classification.

### Statistical analysis

We used EZR (Saitama Medical Center, Jichi Medical University, Saitama, Japan), which is a graphical user interface for R (The R Foundation for Statistical Computing, Vienna, Austria) for all analyses. We performed Pearson’s chi-square (χ^2^) and Fisher’s exact probability tests for categorical variables. For unpaired continuous variables, Student’s t-test and Mann–Whitney’s U test were performed to compare clinicopathological characteristics between the two groups. Survival curves were estimated using the Kaplan–Meier method, and the log-rank test was used to assess statistical differences. The data were stratified for the multivariate analysis by using forward and backward stepwise Cox regression methods. *P* < 0.05 was considered statistically significant^40^.

### Ethical approval

This study was institutionally approved by Kyoto Prefectural University of Medicine.

## Supplementary Information

Supplementary Information 1.

Supplementary Information 2.

Supplementary Information 3.

Supplementary Information 4.

Supplementary Information 5.

## Data Availability

The datasets generated and/or analysed during the current study are not publicly available due to the personal information protection law in Japan but are available after the permission from the institutional review board and the corresponding author on reasonable request.
