# Supplementary material for: Obesity paradox as a new insight from postoperative complications in gastric cancer
Source: Sci Rep. 2023 Jun 21;13:10116. doi: 10.1038/s41598-023-36968-7 (PMC10284837; doi:10.1038/s41598-023-36968-7)
Supplement: Supplementary file 1 — Supplementary Information 1. [file 41598_2023_36968_MOESM1_ESM.pptx]

## Slide 1
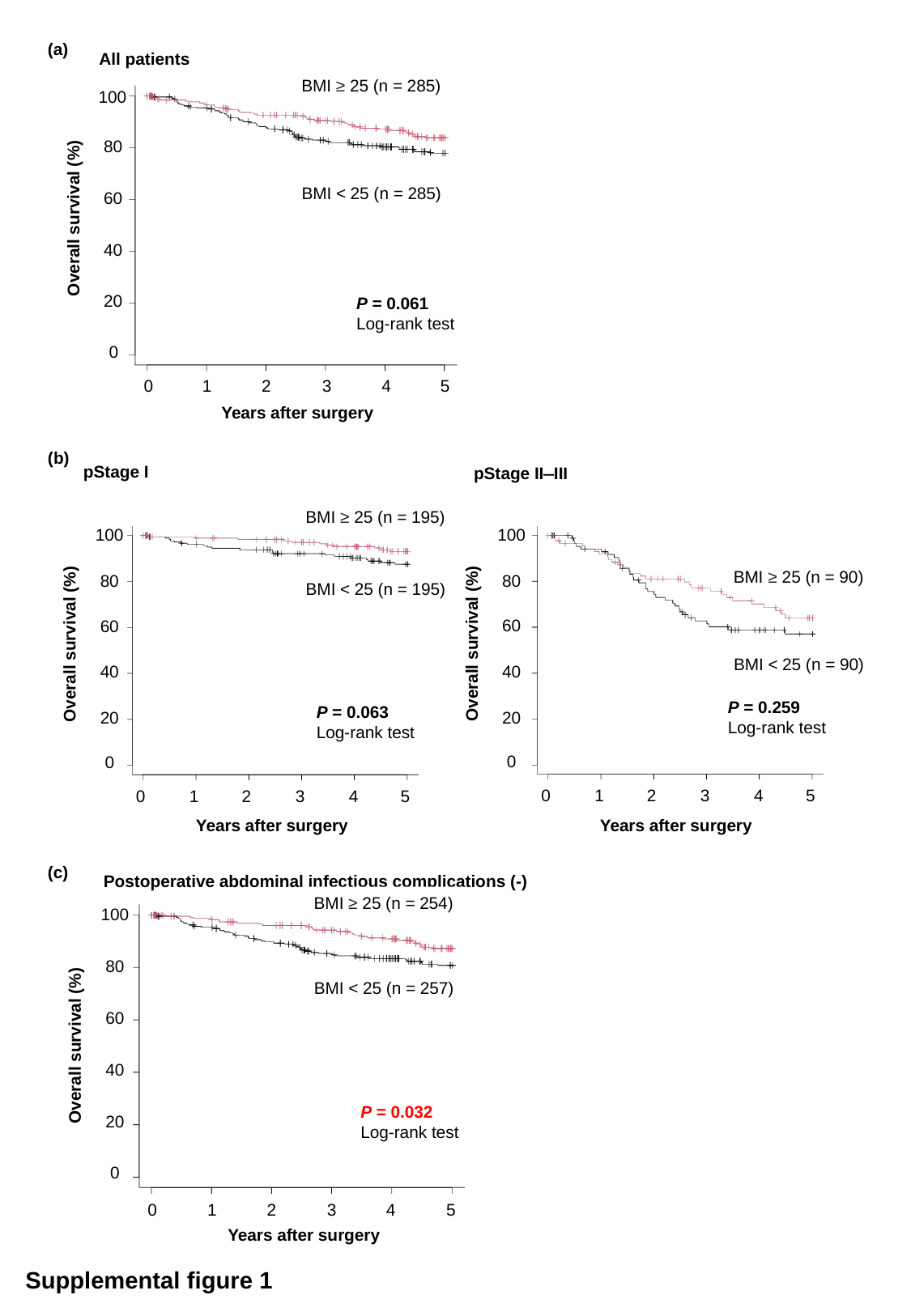

(a)
All patients
BMI ≥ 25 (n = 285)
100
80
BMI < 25 (n = 285)
60
Overall survival (%)
40
20
P = 0.061
Log-rank test
0
0
1
2
3
4
5
Years after surgery
(b)
pStage I
pStage II⎼III
BMI ≥ 25 (n = 195)
100
100
BMI ≥ 25 (n = 90)
80
80
BMI < 25 (n = 195)
60
60
Overall survival (%)
Overall survival (%)
BMI < 25 (n = 90)
40
40
P = 0.259
Log-rank test
P = 0.063
Log-rank test
20
20
0
0
0
1
2
3
4
5
0
1
2
3
4
5
Years after surgery
Years after surgery
(c)
Postoperative abdominal infectious complications (-)
BMI ≥ 25 (n = 254)
100
80
BMI < 25 (n = 257)
60
Overall survival (%)
40
P = 0.032
Log-rank test
20
0
0
1
2
3
4
5
Years after surgery
Supplemental figure 1

## Slide 2
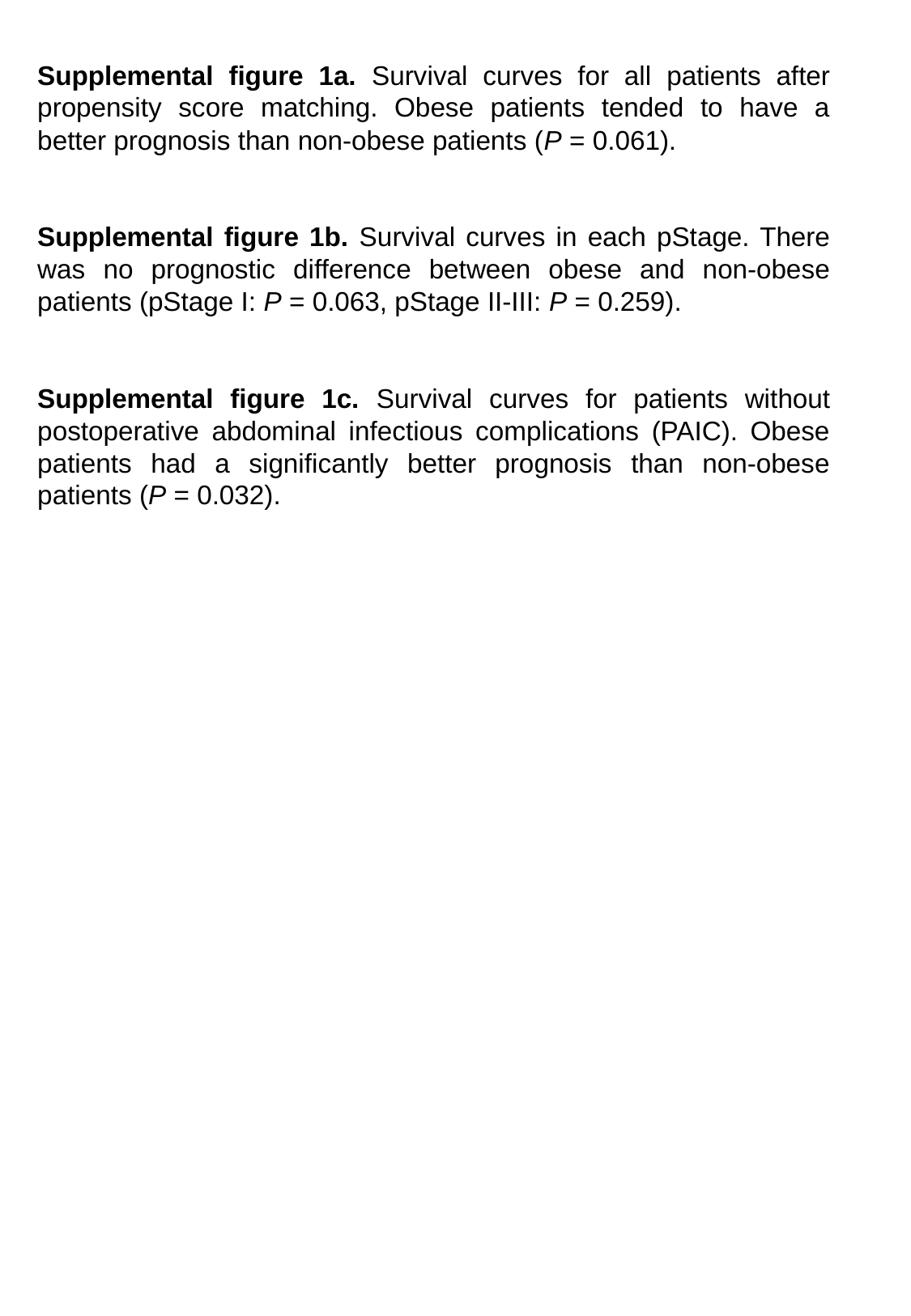

Supplemental figure 1a. Survival curves for all patients after propensity score matching. Obese patients tended to have a better prognosis than non-obese patients (P = 0.061).
Supplemental figure 1b. Survival curves in each pStage. There was no prognostic difference between obese and non-obese patients (pStage I: P = 0.063, pStage II-III: P = 0.259).
Supplemental figure 1c. Survival curves for patients without postoperative abdominal infectious complications (PAIC). Obese patients had a significantly better prognosis than non-obese patients (P = 0.032).
