# Supplementary material for: Obesity paradox as a new insight from postoperative complications in gastric cancer
Source: Sci Rep. 2023 Jun 21;13:10116. doi: 10.1038/s41598-023-36968-7 (PMC10284837; doi:10.1038/s41598-023-36968-7)
Supplement: Supplementary file 2 — Supplementary Information 2. [file 41598_2023_36968_MOESM2_ESM.pptx]

## Slide 1
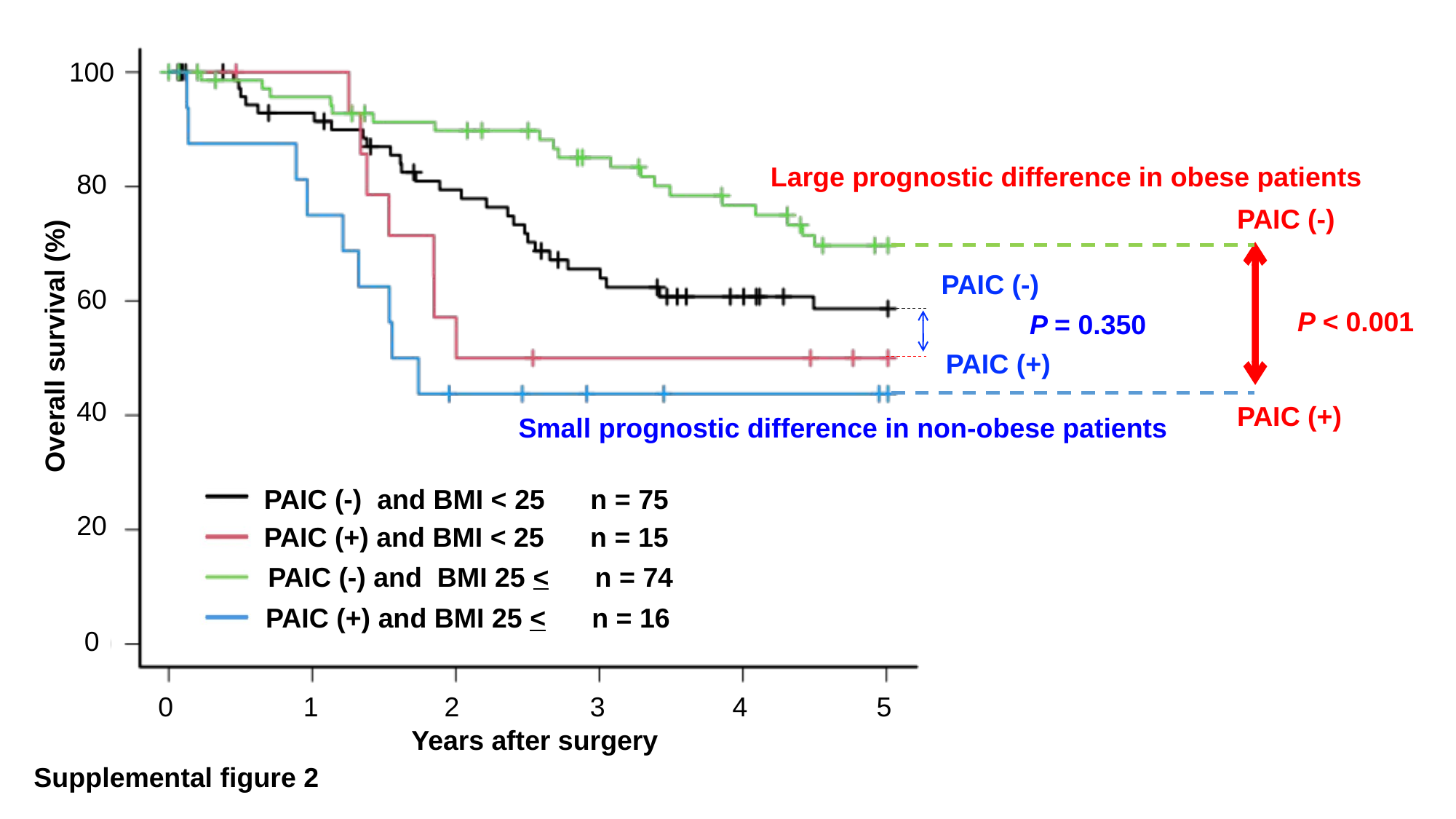

100
Large prognostic difference in obese patients
80
PAIC (-)
PAIC (-)
60
P < 0.001
P = 0.350
Overall survival (%)
PAIC (+)
40
PAIC (+)
Small prognostic difference in non-obese patients
PAIC (-) and BMI < 25 n = 75
PAIC (+) and BMI < 25 n = 15
PAIC (-) and BMI 25 < n = 74
PAIC (+) and BMI 25 < n = 16
20
0
0
1
2
3
4
5
Years after surgery
Supplemental figure 2

## Slide 2
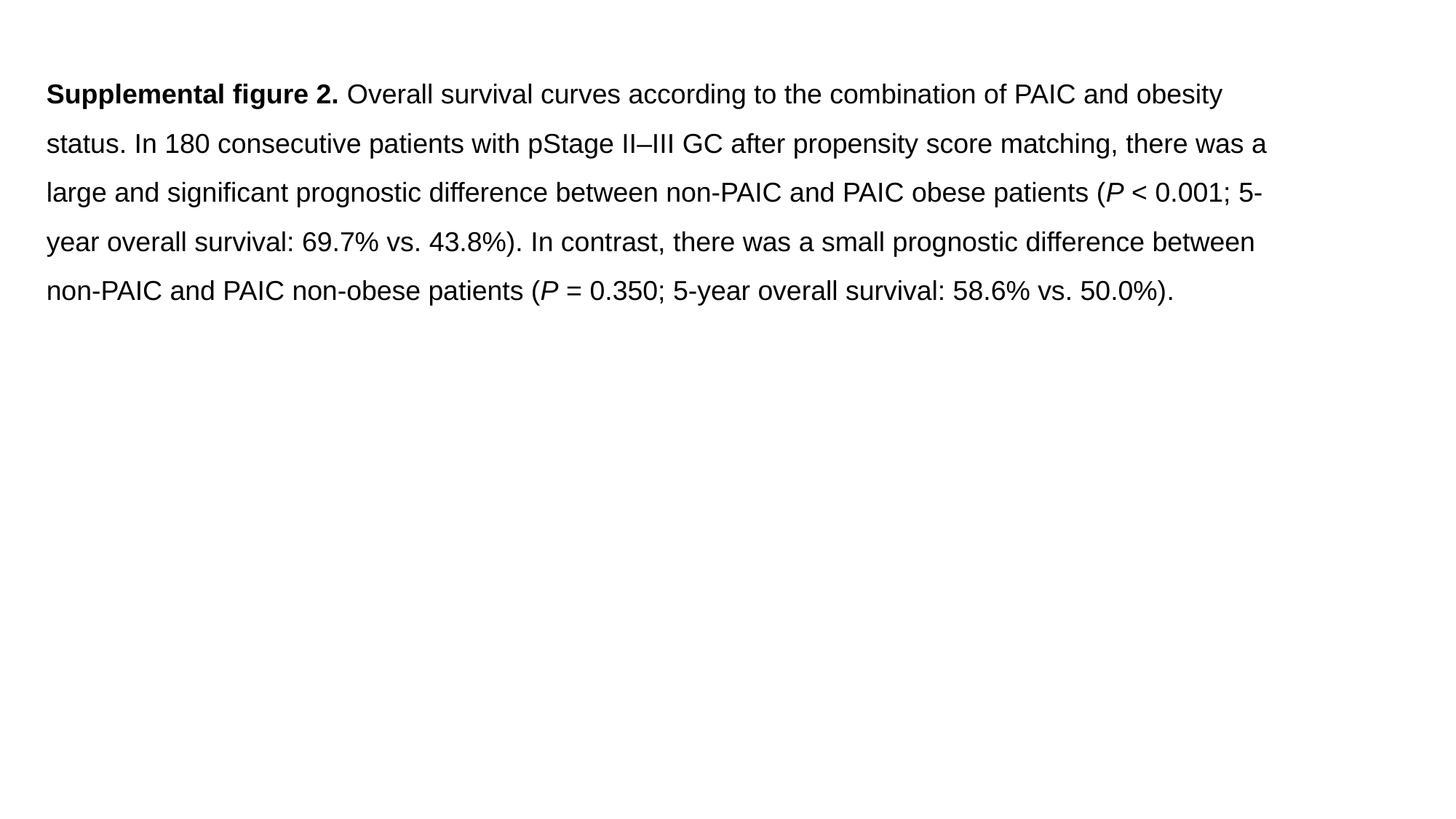

Supplemental figure 2. Overall survival curves according to the combination of PAIC and obesity status. In 180 consecutive patients with pStage II–III GC after propensity score matching, there was a large and significant prognostic difference between non-PAIC and PAIC obese patients (P < 0.001; 5-year overall survival: 69.7% vs. 43.8%). In contrast, there was a small prognostic difference between non-PAIC and PAIC non-obese patients (P = 0.350; 5-year overall survival: 58.6% vs. 50.0%).
