# Supplementary material for: Obesity paradox as a new insight from postoperative complications in gastric cancer
Source: Sci Rep. 2023 Jun 21;13:10116. doi: 10.1038/s41598-023-36968-7 (PMC10284837; doi:10.1038/s41598-023-36968-7)
Supplement: Supplementary file 3 — Supplementary Information 3. [file 41598_2023_36968_MOESM3_ESM.docx]

**Supplemental table 1: Clinicopathological characteristics of obese and non-obese gastric cancer patients after propensity score matching**

| Variables |  | Total | Obese patients  (n = 285) | | Non-obese patients  (n = 285) | | *P*-value |
| --- | --- | --- | --- | --- | --- | --- | --- |
| Gender | Female | 503 | 73 | (26%) | 71 | (25%) | 0.923 |
|  | Male | 1033 | 212 | (74%) | 214 | (75%) |  |
| Age | < 75 | 1194 | 220 | (77%) | 226 | (79%) | 0.612 |
|  | ≥ 75 | 342 | 65 | (23%) | 59 | (21%) |  |
| Albumin (g/dl) | < 3 | 168 | 25 | (9%) | 34 | (12%) | 0.271 |
|  | ≥ 3 | 1368 | 260 | (91%) | 251 | (88%) |  |
| Hemoglobin (g/dl) | < 8 | 115 | 20 | (7%) | 17 | (6%) | 0.734 |
|  | ≥ 8 | 1421 | 265 | (93%) | 268 | (94%) |  |
| Cardiovascular disease | (-) | 1091 | 176 | (62%) | 174 | (61%) | 0.931 |
|  | (+) | 445 | 109 | (38%) | 111 | (39%) |  |
| Digestive disease | (-) | 1137 | 219 | (77%) | 210 | (74%) | 0.437 |
|  | (+) | 399 | 66 | (23%) | 75 | (26%) |  |
| Endocrine and autoimmune disease | (-) | 1285 | 223 | (78%) | 224 | (79%) | 1.000 |
|  | (+) | 251 | 62 | (22%) | 61 | (21%) |  |
| Hepatic disease | (-) | 1442 | 269 | (94%) | 269 | (94%) | 1.000 |
|  | (+) | 94 | 16 | (6%) | 16 | (6%) |  |
| Neurological disease | (-) | 1460 | 277 | (97%) | 276 | (97%) | 1.000 |
|  | (+) | 76 | 8 | (3%) | 9 | (3%) |  |
| Renal and urologic disease | (-) | 1498 | 281 | (99%) | 276 | (97%) | 0.261 |
|  | (+) | 38 | 4 | (1%) | 9 | (3%) |  |
| Respiratory disease | (-) | 1472 | 275 | (97%) | 277 | (97%) | 0.812 |
|  | (+) | 64 | 10 | (3%) | 8 | (3%) |  |
| pStage | I | 1037 | 195 | (68%) | 195 | (68%) | 1.000 |
|  | II + III | 499 | 90 | (32%) | 90 | (32%) |  |
| Surgical approach | Open | 1051 | 198 | (70%) | 181 | (64%) | 0.156 |
|  | Lap | 485 | 87 | (30%) | 104 | (36%) |  |
| Lymphadenectomy | D2 ≤ | 707 | 126 | (44%) | 135 | (47%) | 0.501 |
|  | < D2 | 829 | 159 | (56%) | 150 | (53%) |  |
| PAIC^a^ | (-) | 1396 | 254 | (89%) | 257 | (90%) | 0.784 |
|  | (+) | 140 | 31 | (11%) | 28 | (10%) |  |
| ^a^ Postoperative abdominal infectious complications: anastomotic leakage, pancreatic fistula, and intra-abdominal abscess in grade II or higher of Clavien–Dindo classification. | | | | | | | |
