# Supplementary material for: Obesity paradox as a new insight from postoperative complications in gastric cancer
Source: Sci Rep. 2023 Jun 21;13:10116. doi: 10.1038/s41598-023-36968-7 (PMC10284837; doi:10.1038/s41598-023-36968-7)
Supplement: Supplementary file 4 — Supplementary Information 4. [file 41598_2023_36968_MOESM4_ESM.docx]

**Supplemental table 2: Multivariate analyses of survival after surgery using the Cox’s proportional hazard model according to obesity status after propensity score matching**

|  |  |  |  |  | Obese patients | | |  |  |  | Non-obese patients | | |  |
| --- | --- | --- | --- | --- | --- | --- | --- | --- | --- | --- | --- | --- | --- | --- |
|  |  |  |  |  | Multivariate ^a^ | | |  |  |  | Multivariate ^a^ | | |  |
|  |  |  |  | HR ^b^ | 95% CI ^c^ | | | *P*-value |  | HR ^b^ | 95% CI ^c^ | | | *P-*value |
| Age | 75 ≤ | vs. | < 75 |  |  |  |  |  |  |  |  |  |  |  |
| pStage | II + III | vs. | I | 5.99 | 2.96 | - | 12.12 | < 0.001 |  | 4.86 | 2.57 | - | 9.21 | < 0.001 |
| Surgical approach | Open | vs. | Lap |  |  |  |  |  |  | 5.28 | 2.28 | - | 12.21 | < 0.001 |
| Lymphadenectomy | D2 ≤ | vs. | < D2 |  |  |  |  |  |  | 3.15 | 1.66 | - | 6.00 | < 0.001 |
| PAIC ^d^ | (+) | vs. | (-) | 4.22 | 2.11 | - | 8.44 | < 0.001 |  | 2.54 | 1.31 | - | 4.92 | 0.005 |
| ^a^ Multivariate survival analysis was performed using Cox’s proportional hazard model. | | | | | | | | | | | | | | |
| ^b^ HR: Hazard ratio | | | | | | | | | | | | | | |
| ^c^ CI: Confidence interval | | | | | | | | | | | | | | |
| ^d^ Postoperative abdominal infectious complications: anastomotic leakage, pancreatic fistula, and intra-abdominal abscess in grade II or high of Clavien–Dindo classification. | | | | | | | | | | | | | | |
