# Supplementary material for: Obesity paradox as a new insight from postoperative complications in gastric cancer
Source: Sci Rep. 2023 Jun 21;13:10116. doi: 10.1038/s41598-023-36968-7 (PMC10284837; doi:10.1038/s41598-023-36968-7)
Supplement: Supplementary file 5 — Supplementary Information 5. [file 41598_2023_36968_MOESM5_ESM.docx]

**Supplemental table 3: Recurrence patterns and rates in patients with pStage II–III gastric cancer according to the combination of obesity and PAIC after propensity score matching**

| Recurrence | Obesity (+) PAIC (+)  (n = 16) | Obesity (+) PAIC (-)  (n = 74) | *P*-value | Obesity (-) PAIC (+)  (n = 15) | Obesity (-) PAIC (-)  (n = 75) | *P*-value |
| --- | --- | --- | --- | --- | --- | --- |
| Total patients | 7 (44%) | 22 (30%) | 0.376 | 6 (40%) | 28 (37%) | 0.774 |
|  |  |  |  |  |  |  |
| Recurrence types |  |  |  |  |  |  |
| Peritoneum | 5 (31%) | 7 (10%) | **0.035** | 2 (13%) | 15 (20%) | 0.713 |
|  |  |  |  |  |  |  |
| Local | 1 (6%) | 2 (3%) | 0.448 | 0 (0%) | 4 (5%) | 1.000 |
|  |  |  |  |  |  |  |
| Lymph node | 1 (6%) | 7 (10%) | 1.000 | 1 (6.7%) | 8 (11%) | 1.000 |
|  |  |  |  |  |  |  |
| Hematogenous | 2 (13%) | 10 (14%) | 1.000 | 4 (26.7%) | 8 (11%) | 0.110 |
|  |  |  |  |  |  |  |
| Others | 2 (13%) | 1 (1%) | 0.326 | 0 (0.0%) | 2 (3%) | 1.000 |
|  |  |  |  |  |  |  |
